# Marine Drug Research in Italy

**DOI:** 10.3390/md24100353

**Published:** 2026-10-08

**Authors:** Stella Cascioferro

**Affiliations:** Department of Biological, Chemical and Pharmaceutical Sciences and Technologies (STEBICEF), University of Palermo, 90123 Palermo, Italy; stellamaria.cascioferro@unipa.it

The extraordinary structural diversity of marine natural products, together with their broad range of biological activities, has made the marine environment an increasingly important source of inspiration for drug discovery [1]. Over the past decades, numerous marine-derived natural products were used as valuable starting points for the discovery of new therapeutic agents [2,3,4]. In this rapidly evolving field, Italy is recognized as a research community with a strong international standing, supported by rich marine biodiversity and a long-standing tradition in natural product chemistry [5,6].

The special issue of Marine Drugs, titled “Marine Drug Research in Italy” (https://www.mdpi.com/journal/marinedrugs/special_issues/NVNKS2BI42) (accessed on 27 September 2026) aims to highlight the growing interest of Italian research in this field. The seven articles collected in this Special Issue, including five original research articles and two reviews, provide a representative snapshot of current Italian research activities on marine bioactive compounds. The individual works are listed in the List of Contributions and discussed below.

In contribution 1 (doi:10.3390/md22020075) Fontana et al. explores the chemical composition and the biological properties of the marine ascidians *Cystodytes dellechiajei* collected in Cartaromana Bay (Ischia Island, Italy). A bio-guided investigation led to the identification of two pyridoacridine alkaloids, shermilamine B and N-deacetylshermilamine B, along with a family of methyl-branched cerebrosides. In particular, N-deacetylshermilamine B proved to be a a selective and dose-dependent ligand of TREM2, a receptor with an important role in innate immune regulation and in neurodegenerative diseases. This finding is particularly noteworthy, as N-deacetylshermilamine B represents the first alkaloid reported to interact with TREM2, highlighting its potential as a promising lead compound for the development of novel TREM2-targeted modulators. Additionally, both N-deacetylshermilamine B and shermilamine B exhibited promising cytotoxic activity against the myeloid cancer cell lines JJN-3 and KMS-12. The combination of the immunomodulatory and cytotoxic activity found in N-deacetylshermilamine B (2) may suggest its potential development as an inducer of immunogenic cell death.

Contribution 2 (doi:10.3390/md22030133) described the design, synthesis, and biological evaluation of a new series of [1,3,4]thiadiazole[3,2-*a*]pyrimidin-5-ones, obtained through a molecular hybridization approach combining key structural features of the marine alkaloid nortopsentin and the triazolopyrimidine antibiotic Essramycin. The new compounds displayed remarkable antibiofilm activity against a broad range of clinically relevant Gram-positive and Gram-negative pathogens, including *Staphylococcus aureus* ATCC 25923, *Pseudomonas aeruginosa* ATCC 15442, and *Escherichia coli* ATCC 25922. Notably, since the derivatives exhibited both ability to inhibit biofilm formation and to disperse preformed biofilms, without affecting microbial viability, they showed a typical anti-virulence profile. Among the synthesized compounds, the most promising derivative showed significant biofilm-dispersing effects also against the fungus *Candida albicans*. Its anti-infective potential was further investigated in vivo using the *Galleria mellonella* model, where the thiadiazopyrimidinone exhibited a favorable toxicity profile and promising protective activity.

In contribution 3 (doi:10.3390/md22080350) Ostacolo et al. provided a comprehensive overview of the pharmacological modulation of voltage-gated potassium K^+^ channels by marine toxins and marine-derived compounds. The review highlighted the remarkable structural and pharmacological diversity of these molecules, which can interact with different ion-channel subtypes with various degrees of potency and selectivity. Particular attention was given to the evaluation of their mechanisms of action and to their potential as molecular tools for investigating ion-channel function and as starting points for drug discovery. Given the central role of voltage-gated K^+^ channels in neuronal excitability and other physiological processes, their modulation may offer therapeutic opportunities in several pathological conditions, including pain, neurological disorders, and autoimmune diseases. Overall, this contribution underscored the potential of marine toxins as inspiration for the development of novel ion-channel-targeting therapeutics.

In contribution 4 (doi:10.3390/md22090380) Iseppi et al. explored the antimicrobial potential of hallachrome, an unusual anthraquinone produced by the Mediterranean marine polychaete *Halla parthenopeia*. Hallachrome was identified as a major component of the purple mucus released by the worm under stressful conditions, supporting its proposed role in chemical defense. The compound exhibited pronounced toxicity against a broad range of microorganisms showing a marked selectivity against Gram-positive bacteria and the fungal pathogen *Candida albicans*. Mechanistic studies highlighted a dose-dependent growth inhibition exerted by altering the membrane permeability of the pathogens studied. Additionally, hallachrome exhibited significant antibiofilm activity, markedly reducing biofilm formation and disrupting established biofilm structures in several relevant pathogens, including *S. aureus*, *E. faecalis*, and *C. albicans*.

Contribution 5 (doi:10.3390/md22110511) reported the evaluation of the invasive macroalga *Chaetomorpha linum* as a sustainable source of antimicrobial compounds within a circular bioeconomy framework. The study focused on biomass collected from the Orbetello Lagoon (Tuscany, Italy), where the extensive growth of this macroalga represents an environmental and economic challenge and requires regular mechanical harvesting. Phytochemical analysis of a *C. linum* extract identified palmitic acid as the predominant bioactive component, while microbiological assays demonstrated notable antibacterial activity, particularly against *Enterococcus faecalis*. In addition, in silico analyses provided insights into the potential interaction of palmitic acid with a molecular target in *E. faecalis,* the protein EbpA. Overall, this study exemplifies how the valorization of an invasive marine biomass can simultaneously address an environmental challenge and provide a sustainable source of antimicrobial compounds, illustrating the potential of circular approaches in marine drug discovery.

Contribution 6 (doi:10.3390/md22110523) provided a comprehensive overview of the effects of marine-derived components on cardiovascular disease risk factors and gut microbiota diversity. The review examined a broad range of bioactive compounds, including omega-3 polyunsaturated fatty acids, fish-derived proteins and peptides, algal components, polyphenols, carotenoids, and other secondary metabolites, highlighting their potential to modulate key cardiovascular risk factors such as lipid metabolism, blood pressure, oxidative stress, inflammation, and endothelial function. Particular attention was given to the interplay between these marine-derived dietary components and the gut microbiota, with a focus on their ability to influence microbial composition and metabolic activity and, consequently, pathways involved in cardiovascular health.

Contribution 7 (doi:10.3390/md23010045) described the potential of the exoskeleton of the invasive blue crab *Callinectes sapidus* as a source of bioactive molecules, such as chitosan, astaxanthin, and bio-phenolic compounds, with antioxidant and antitumor properties. The extracts obtained from the crab exoskeleton, showed reducing and radical-scavenging activities, providing evidence of their potential to counteract oxidative processes. Additionally, the astaxanthin and polyphenolic extracts also exhibited antiproliferative activity against the cancer cell lines HepG2 and CaCo-2. The results described stem from growing interest in sustainable approaches aimed at recovering marine-derived molecules with potential applications in the pharmaceutical and nutraceutical sectors.
